# Myolipoma of Mesentery: A Case Report

**DOI:** 10.1155/2013/823823

**Published:** 2013-12-26

**Authors:** Hyun Sung Kim, Suk Kim, Kyungbin Kim, Kyung Un Choi, Joo Youn Kim

**Affiliations:** ^1^Department of Surgery, Pusan National University Hospital, Busan 602-739, Republic of Korea; ^2^Department of Radiology, Pusan National University Hospital, School of Medicine, Pusan National University, Yangsan-si 626-770, Republic of Korea; ^3^Department of Pathology, Pusan National University Yangsan Hospital, Yangsan-si 626-770, Republic of Korea; ^4^Department of Pathology, School of Medicine, Pusan National University, Yangsan-si 626-770, Republic of Korea; ^5^Department of Pathology, Pusan National University Hospital, Busan 602-739, Republic of Korea

## Abstract

Myolipomas are very rare benign lipomatous soft tissue tumors which are usually located in retroperitoneum, abdominal and pelvic cavity, and the abdominal wall. They can be diagnosed histologically by the presence of irregularly admixed mature adipose tissue and smooth muscle fibers. The correct diagnosis of myolipoma is important, because it should be considered in the differential diagnosis of fat-containing lesions of the soft tissue and should follow a benign clinical course despite its frequently large size and deep location. We report here a case of myolipoma arising in the mesentery of the jejunum.

## 1. Introduction

Myolipomas are benign lipomatous soft tissue tumors, first described by Meis and Enzinger in 1991 [1]. These tumors are composed of irregularly admixed mature adipose tissue and smooth muscle fibers. Meis and Enzinger proposed that the soft tissue tumors with mixed mature adipose and smooth muscle tissues should be called “myolipomas of soft tissue” and recognized as a new disease entity. The tumor is very rare. To our knowledge, there have been 25 previously reported cases. Myolipoma occurs most frequently in adults, presents as a single mass, and is usually located in retroperitoneum, abdominal and pelvic cavity, and the abdominal wall [1–8]. We describe here a case of myolipoma arising in the mesentery of the jejunum.

## 2. Case Report

**Figure 1:**
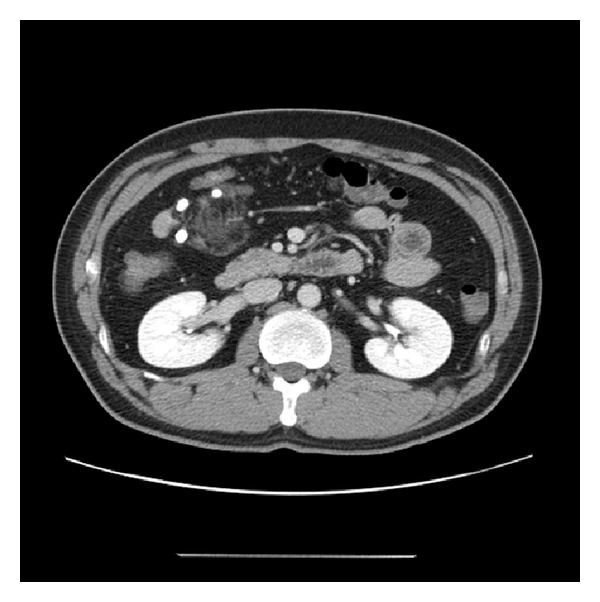
Axial contrast-enhanced CT scan. It shows the tumor measuring 4 cm in size and having fat component in the mesentery.

A 42-year-old male was admitted to our hospital with the main complaint of an abdominal mass, which was incidentally detected on a routine medical checkup. His laboratory studies were within normal limits and his past history was unremarkable. Computed tomography (CT) images showed a 4 cm sized mass. Axial contrast-enhanced CT scan showed a well-demarcated fatty mass lesion containing soft tissue attenuation in the mesentery of the jejunum (Figure 1). The differential diagnosis included sclerosing mesenteritis, lipoma, liposarcoma, and vascular malformation, such as hemangioma. The patient underwent surgery. At operation, a large mesenteric mass was found to arise from the mesentery and adherent to the jejunum. Segmental resection of the jejunum with the mesenteric mass was performed.

**Figure 2:**
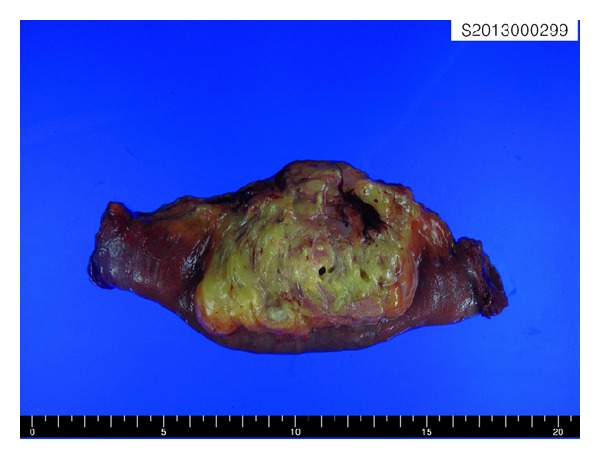
Gross appearance of the tumor. The cut surface of the tumor is yellowish, well demarcated, and partially encapsulated and shows small nodules of firm white tissue, corresponding to smooth muscle. The tumor does not invade the jejunal wall.

**Figure 3:**
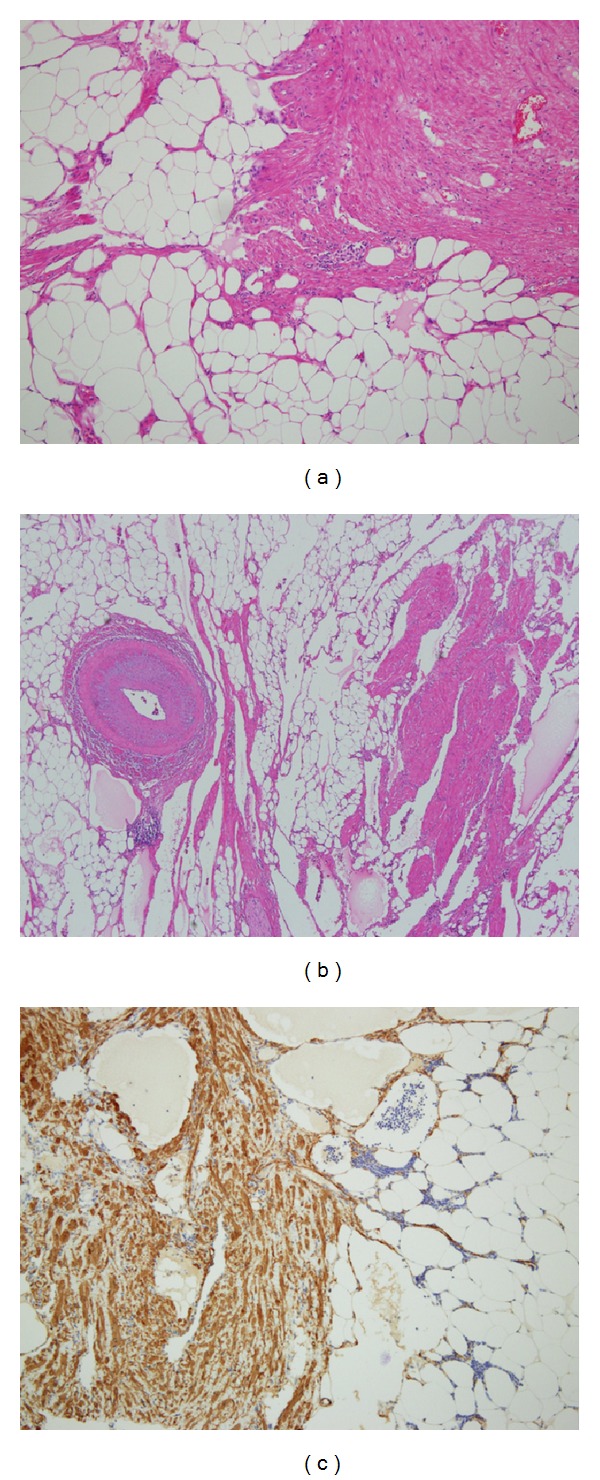
Microscopic findings of the tumor. (a) The tumor shows admixture of smooth muscle and mature adipocytes. (b) Widely dilated vessels and a thick wall vessel are seen within the tumor. (c) Smooth muscle cells are immunoreactive with alpha-smooth muscle actin.

The mass, which measured approximately 5.5 × 5.4 × 4.8 cm, was elliptical in shape with a partial thin capsule. The cut surface was solid and mostly made up of the yellowish soft tissue admixed with a foci of the whitish, firm tissue. It was softer than a leiomyoma and firmer than a lipoma. There were no areas of hemorrhage or necrosis (Figure 2). Microscopically, the tumor consists of a mix of mature adipose tissue and bundles of the spindle cells, which had features of mature smooth muscle cells with regular cigar-shaped nuclei and bright fibrillary eosinophilic cytoplasm (Figure 3(a)). The fat component predominanted, with a fat to muscle ratio of approximately 4 : 1, although the two components were intermingled with each other. Characteristically, abundant dilated blood vessels were observed and a few thick walled vessels are also noted (Figure 3(b)). Mitoses, necrosis, and cytologic atypia were absent. Immunohistochemically, the adipocytes were found to be positive for S100 protein and the smooth muscle cells were diffusely positive for the alpha-smooth muscle actin and desmin. No tumor cells were positive for melanoma markers, including HMB45 and Melan-A, as well as c-kit and CD34. These findings led us to diagnose the lesion as a myolipoma of the soft tissue.

The patient was discharged a week after surgery without a significant postoperative complication. We carried out the follow-ups regularly for 6 months, and he showed no evidence of recurrence.

## 3. Discussion

Myolipoma is an extremely rare benign tumor, which is composed of variable amounts of benign smooth muscle and mature adipose tissue. Myolipoma of soft tissue has been introduced for the first time as a new entity in the 2002 World Health Organization (WHO) Classification of Tumors of Soft Tissue and Bone as part of adipocytic tumors [9]. In the uterus, similar tumors are known as lipoleiomyomas, the term that some use when referring to the myolipoma of soft tissue [2]. Therefore, WHO classification defines that myolipoma of soft tissue is a benign extrauterine tumor composed of the mature adipose tissue and smooth muscle.

Myolipoma occurs frequently in adult (ranged from 4 to 83, median 52), with a predominantly predilection for women. It was usually located in the retroperitoneum [1, 3–8], the abdominal and pelvic cavity [1, 8], and the abdominal wall [1, 2]. Other locations have been reported such as the subcutaneous tissue [1], intramuscular region [10], and inguinal canal [1]. There is no report about involvement of visceral organ. They are 3.5–30 cm in size. Some cases were clinically palpable mass and the other cases were incidental findings or various symptoms according to the anatomic sites, such as abdominal distension [5].

Macroscopically, the tumor is either partially or completely encapsulated, and the cut surface demonstrates a yellow to white cut surface. Histologically, myolipoma is composed of an irregular admixture of mature adipocytic tissue and bundles and sheets of well-differentiated smooth muscle in varying proportions. The tumor lacks atypia and mitoses and displays little vascular proliferation. The medium-caliber arteries with thick muscular walls were absent in most cases. Characteristically, the case reported here showed a few thick walled vessels and numerous vessels with the large widely dilated lumens. Michal [3] reported two similar retroperitoneal tumors as myolipomas and emphasized the presence of dilated nonneoplastic vascular structures in the tumors. The medium-caliber arteries with thick muscular walls were present in cases occurring in the mesenteric root [1].

The differential diagnosis of myolipoma includes well-differentiated liposarcoma, dedifferentiated liposarcoma, spindle cell lipoma, angiomyolipoma, omental infarction, hamartoma, and leiomyoma with fatty degeneration. Myolipomas are often misdiagnosed clinically and radiologically as liposarcoma, especially, if the tumor is deeply located, such as in retroperitoneum. Myolipoma can be distinguished from liposarcoma because there is at least partial encapsulation, absence of mitoses with no atypia, and no lipoblasts. Spindle cell lipoma was also considered in the differential diagnosis. The presence of the smooth muscle, the anatomic site, and the deep location exclude that diagnosis. Myolipoma differs from angiomyolipoma. Angiomyolipoma shares the huge size and occasional location in the retroperitoneum with myolipoma. In our case, microscopic findings closely mimicked those of an angiomyolipoma because of abundance in vessels. However, the lack of abundant thick walled blood vessels and infiltrating borders as well as the lack of the immunoreactivity of the smooth muscle component for HMB45 argues against angiomyolipoma.

Myolipoma is a distinct entity that appears to follow a benign clinical course despite its frequently large size and deep location. There are no reports of local recurrence, metastatic disease, or malignant transformation, although follow-up data were available in the limited cases (range, 1–120 months; median, 10.5) [1, 3, 8]. Surgical excision is the treatment of choice for these lesions.

In conclusion, we reported the rare case of soft tissue myolipoma in the mesentery. Despite the benign nature of these tumors, the correct diagnosis is important, because such masses need to be considered in the differential diagnosis of fat-containing lesions of the soft tissue.
